# Comprehensive investigation of alternative splicing and development of a prognostic risk score for prostate cancer based on six-gene signatures

**DOI:** 10.7150/jca.31725

**Published:** 2019-09-07

**Authors:** Zhe-Xu Cao, Guang-An Xiao, Wei Zhang, Jin Ji, Chen Ye, Dan Liu, Qin-Qin Tian, Ying-Hao Sun Prof

**Affiliations:** 1 Department of Urology, Shanghai Changhai Hospital, Naval Medical University (Second Military Medical University), Shanghai 200433, China.; 2 Shanghai Key Laboratory of Cell Engineering, Naval Medical University (Second Military Medical University), Shanghai 200433, China.

**Keywords:** prostate cancer, prognosis, alternative splicing, TCGA

## Abstract

**Purpose**: To systematically document alternative splicing profiles of prostate cancer in relatively large populations in order to construct a prognostic predictors model for prostate cancer.

**Methods**: Splicing data and clinical information of 495 prostate cancer patients were obtained from The Cancer Genome Atlas (TCGA). The SpliceSeq database was used to extract information regarding splicing events. Multiple bioinformatic tools were used for functional and pathway enrichment analysis as well as for construction of gene interaction networks. Candidate gene expression profiles were verified with clinical samples using QRT-PCR.

**Results**: We detected a total of 44070 alternative splicing events of 10381 genes in prostate cancer. 7 and 14 KEGG pathways were enriched and were associated with overall and recurrence-free survival, respectively. The expression of 396 genes among the 1526 overall survival genes associated alternative splicing events were associated with overall survival. The expression of 483 genes among the 1916 recurrence-free survival genes associated alternative splicing events were associated with recurrence-free survival. Lastly, we constructed the prognosis risk score system based on the expression profiles of six-gene signatures which in combination had an AUC of 0.941 for overall survival associated alternative splicing events, followed by overall survival associated gene expressions with an AUC of 0.794, a recurrence-free survival associated gene expression with an AUC of 0.752 and recurrence-free survival associated alternative splicing events with an AUC of 0.735, indicating its strong ability to predict patient outcome. The expression profile of the six genes was also confirmed in different prostate cell lines and clinic samples.

**Conclusion**: Our comprehensive investigation of alternative splicing not only provided insight into the biological pathways of alternative splicing involved in the development of prostate cancer but also revealed new potential biomarkers for prognosticating as well as novel therapeutic targets for development of prostate cancer treatment.

## Introduction

Alternative splicing (AS) is a naturally occurring event that serves to regulate the volume of functional mRNA. It contributes towards the proteome diversity in a given organism by increasing the complexity of the transcriptome. More than 90% of human genes are known to have alternative forms of splicing, with about 60% of these splice variants encoding distinct protein isoforms. Through the production of a broad range of protein species, AS confers an evolutionary advantage for more complex life forms. Nevertheless, AS dysfunction leads to aberrant proteins, which has been thought to lead to the development of several human diseases, including cancer 1.

Prostate cancer (PCa) accounts for almost one in five of all newly diagnosed cancer and is projected to be the most frequent cancer amongst men in 2018 (164,690 new cases). Moreover, estimated deaths of PCa occupied the second (29,430 deaths) amongst men 2. Increasing evidence demonstrates that AS is involved in the initiation and progression of cancer. Several splice variants that are known to be involved in cancer have been identified in PCa, including VEGFA, KLF6, BCL2L2, ERG, FGFR2, TMPRSS2-ERG and AR 3,4. Therefore, AS disorders are to be recognized as important biological occurances that can be detected for tumor diagnosis as well as function as potential targets in drug development 5.

AS could also be a potential prognostic factor in PCa. The prognostic value of AS events and patient prognosis have been reported in several cancer types, including lung 6, colon 7 and bladder 8. However, previous studies only focused on the role of a single AS in PCa 9,10,11. Limited efforts have been made in systematic cancer prognostic analyses of AS in PCa patients. Given the considerable amount of AS occurances in PCa, it is entirely feasible that there are several more yet to be discovered AS in this condition.

The Cancer Genome Atlas (TCGA), an initiative spearheaded by the US National Institutes of Health, is home to biological information on samples of more than 10,000 patients with over 33 different types of cancer, and is a rich source of data for investigating patterns of AS in malignancies. SpliceSeq is a resource for RNA-Seq data from TCGA and functions as an easily accessible source of AS and splicing events identification with analysis regarding potential functional changes resulting from splice variations 12. Kahles et al. comprehensively analyzed AS across 32 cancer types from 8,705 patients in order to detect tumor variants and AS through RNA analysis and whole-exome sequencing 13.

Given the importance of AS and the lack of a comprehensive study regarding AS occurrences in PCa, our study seeks to systematically profile AS in PCa in a relatively large population. More importantly, we aim to construct a prognosticating model for PCa that may serve as a foundation for the drug discovery in PCa.

## Materials and methods

### Identification of AS events in TCGA RNA-seq data

Level 3 RNA-seq data and clinical information from 495 patients were extracted from the TCGA data portal (https://tcga-data.nci.nih.gov/tcga/). AS profiles were generated for each patient using the SpliceSeq tool. A total of 495 patients with survival times of more than 90 days and fully characterized TCGA Splice Seq and RNA-Seq data were included in the present study. We annotated exons using GENCODE (GRCh38 p2 version) (https://www.gencodegenes.org/#) and designated exons as either the first (representing alternative transcriptional start), last (representing alternative transcriptional end), or internal within available transcripts. For each exon, its FPKM (Fragments Per Kilobase per Million mapped reads, a measure of its level of expression by RNAseq) was downloaded and normalized as TPM (Trans Per Million) data. The Percent Spliced In (PSI) value, a value between 0 to 1 that is commonly used to quantify AS occurrences, was calculated for seven types of AS events: Mutually Exclusive Exons (ME), Exon Skip (ES), Alternate Acceptor site (AA) and Alternate Donor site (AD), Retained Intron (RI), Alternate Terminator (AT), Alternate Promoter (AP).

### Identification of AS events associated to prognosis

The association between the AS events and patient overall survival (OS) and recurrence-free survival (RFS) were respectively evaluated by univariate Kaplan-Meier analysis, with the *p-*value threshold designated to be 0.05. Patient samples were categorized into two groups using the PSI median value. A Venn diagram is used to present relationships between AS events associated genes in OS and RFS. For the seven types of AS, UpSet plots were used to depict sets that intersected, as a single gene may have more than 2 splicing events that are each significantly linked to OS. As described by Lex et al., UpSet plots are a novel means of depicting intersecting sets in a more comprehensive and quantitative manner in comparison to Venn diagrams, especially in the case of five or more sets 14. A stepwise multivariate Cox regression including the top 10 genes that were significantly associated with prognosis (*p* <0.05) was applied. These genes were then used to construct the area under the curve (AUC) of the receiver-operator characteristic (ROC) curve for each model. The ability of each AS to predict PCa prognosis was derived from the ROC curve 15.

### Gene network construction and functional enrichment analyses

Further exploration of the interactions between alternatively spliced genes in PCa was carried out via gene network analyses. It was performed via the Search Tool for the Retrieval of Interacting Genes (STRING) database (http://www.string-db.org/), a biological databank of predicted and known protein-protein interactions (PPI) that offers a system-wide view of cellular processes. A confidence score of ≥0.4 was determined as the threshold. Gene networks were created with the Cytoscape (version 3.4.0) 16 program. Pathway enrichment analyses based on the Kyoto Encyclopedia of Genes and Genomes (KEGG) were performed for alternatively spliced genes. The *p*-value <0.05 was established as the threshold for KEGG functional analysis.

### Detection of the relationship between alternatively spliced genes expression and prognosis and screening of prognostic signature

TCGA PCa level 3 RNA-seq derived gene expression levels were subjected to univariate Kaplan-Meier analysis. The Pearson correlation coefficient was calculated for the candidate prognosis associated genes and the AS events identified as above. Genes with |Pearson correlation coefficient| >0.50 were considered to be prognostic signatures.

### Development of a six-gene signature prognostic risk score system

6 candidate prognostic genes were subjected to a multivariate Cox regression analysis in order to establish a prognostic risk score for predicting RFS and OS. This score was developed using a linear combination of the PSI value or gene expression level multiplied by the regression coefficient determined using the multivariate Cox regression model (β) with the following formula which has been used in prior studies 17: Risk score = βgene1*expgene1 + βgene2*expgene2 + … βgene6*expgene6 or Risk score = βgene1*PSIgene1 + βgene2*PSIgene2 + … βgene6*PSIgene6.

Two groups (the low-score and high-score group) of patients were identified according to their respective median risk scores. OS and RFS curves were generated with the Kaplan-Meier method, and two-side log-rank tests were employed to compare the survival time between the low-risk and high-risk groups of patients. ROC curves were generated to test the specificity and sensitivity of the prognostic model by calculating the AUC of the ROC curve.

### Confirmation of gene expression using RT‑PCR

To confirm the expression of the six genes, QRT-PCR was performed on prostate cell lines including normal prostate epithelial: RWPE-1, cells derived from in situ PCa: 22Rv1, and cells derived from metastatic site: DU 145, LNCaP, PC-3, and C4-2B.

PCa samples from Shanghai Changhai Hospital were collected and categorized into high (Gleason score =8,9,10) and low (Gleason score =6,7) grade prostate cancer. These samples were then subjected to QRT-PCR analysis. Ethical approval was obtained from the ethics committee of the hospital prior to sample collection. The Bio-Rad Connet Real-Time PCR platform was used to perform QRT-PCR. The Primer-BLAST online tool (https://www.ncbi.nlm.nih.gov/tools/primer-blast/index.cgi?LINK_LOC=BlastHome) was used to design primers utilized for QRT-PCR analysis. The primer sequences were shown in Supplementary Table 1.

### Statistical analysis

All numerical data was expressed as the mean ± SD of three independent experiments. Categorical data was presented in terms of percentages while continuous data was presented as median (range). The univariate and multivariate Cox regression analyses were employed to identify prognosis- associated alternative splicing events. Correlation between alternative splicing events associated genes expression and prognosis were analyzed with Pearson correlation coefficient. Receiver operating characteristic curves and Kaplan-Meier analysis allowed for evaluation of the clinical significance of genes for building a risk score system in PCa. Statistical significance was determined when *p* <0.05. Results were analyzed based on the SPSS version 19.0 (SPSS, Inc.). Gene networks were depicted using Cytoscape version 3.4.0.

## Results

**Figure 1:**
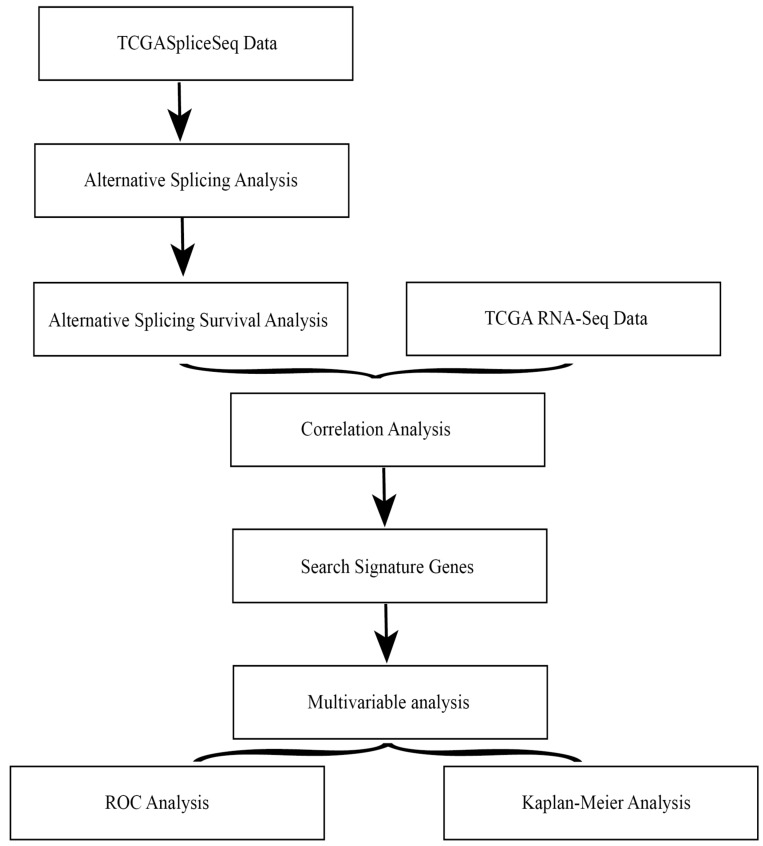
Overall flowchart of the work

The overall flowchart of this work was summarized in Fig. 1.

### Integrated Overview of AS events in TCGA PCa cohort

**Figure 2:**
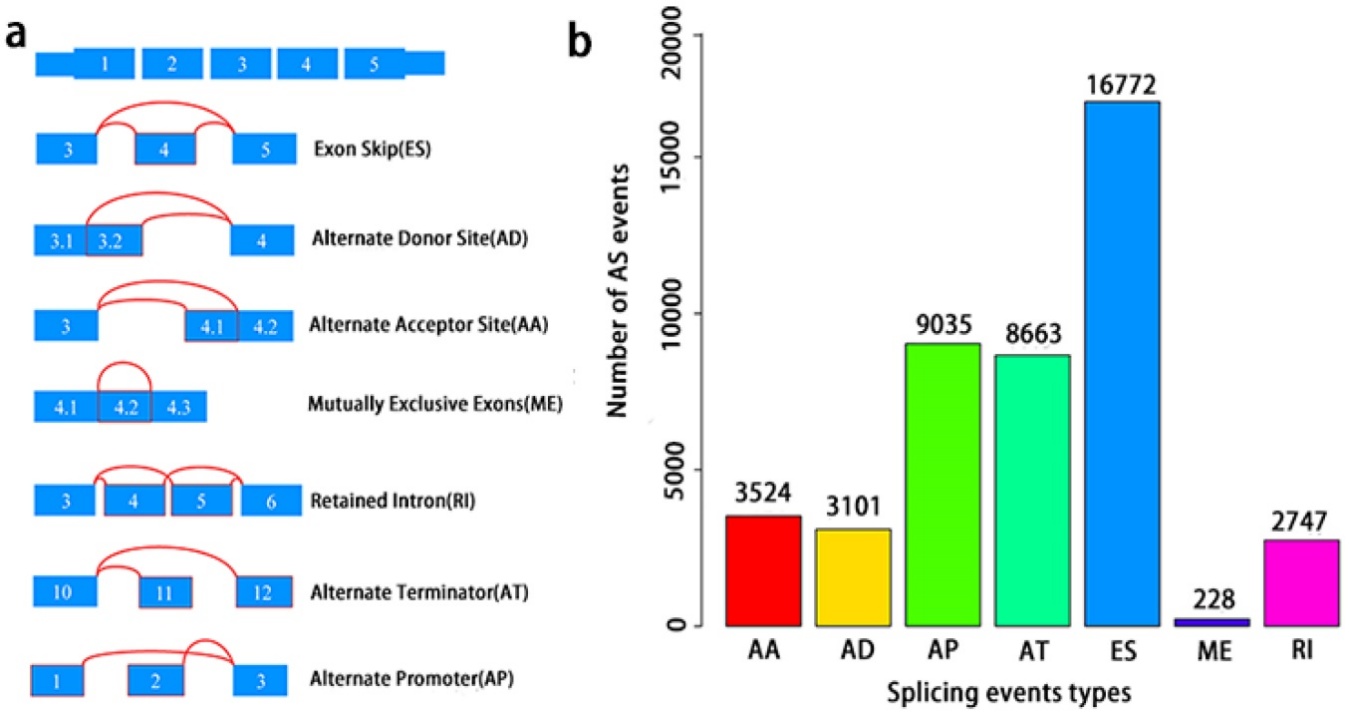
** Overview of seven types of AS in this study.** a, Illustrations for seven types of AS events, including Exon Skip (ES), Alternate Donor site (AD), and Alternate Acceptor site (AA), Retained Intron (RI), Mutually Exclusive Exons (ME), Alternate Terminator (AT), Alternate Promoter (AP). b, Number of AS events and involved genes from the 495 PCa patients.

A total of 495 PCa patients from the TCGA database had their integrated mRNA splicing event profiles analyzed. 10,381 genes revealed 44070 mRNA splicing events which comprised of 228 MEs in 223 genes, 3524 AAs in 2488 genes, 3101 ADs in 2185 genes, 8549 ATs in 3407 genes, 8993 APs in 3304 genes, 2747 RIs in 1851 genes, and 16772 ESs in 6579 genes. These findings hint that several mRNA splicing events may be present in a single gene (Fig. 2a). Up to 50% of these alternative splicing events were ES events, followed by AP and AT events. ME events were rare (Fig. 2b).

### Prognosis-related AS events in the TCGA PCa cohort

**Figure 3:**
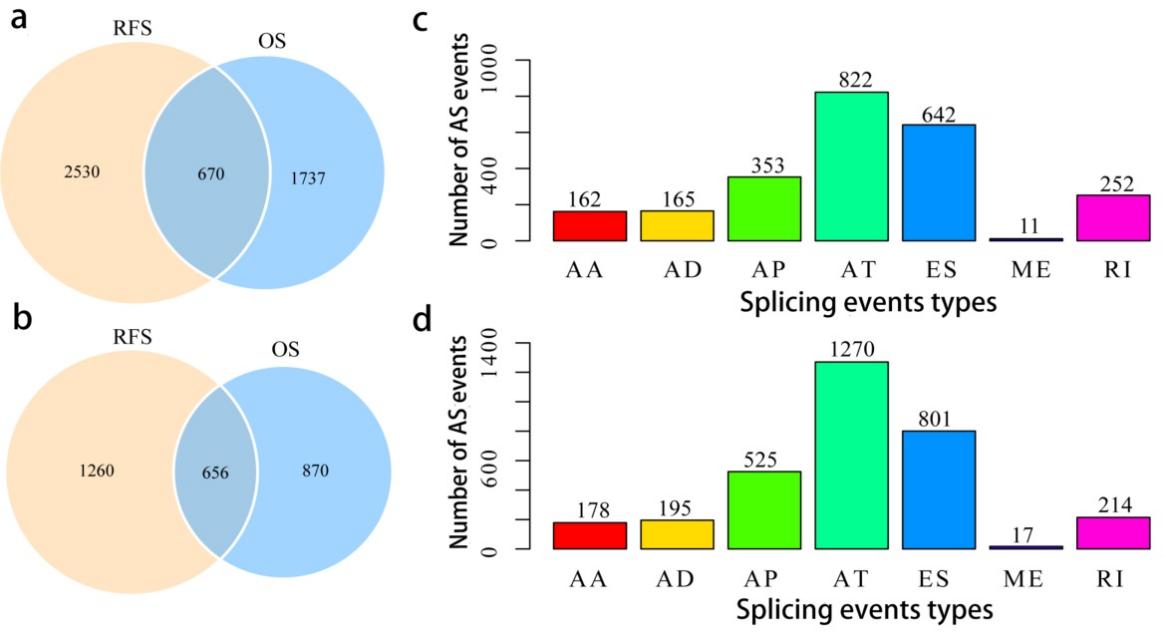
** Overview of prognosis associated AS events in the TCGA PCa cohort.** a, Illustrations for prognosis associated AS events. b, Illustrations for prognosis associated alternatively spliced genes. c, Number of seven types of OS associated AS events. d, Number of seven types of RFS associated AS events.

To assess the prognostic values of splicing events, clinical follow-up data (Supplementary Table 2) was integrated with the PCa patients categorized into either high or low risk groups based on their PSI levels. Univariate survival tests were conducted amongst 44070 splicing events in order to study the relationship between AS events with RFS and OS. A total of 2407 OS associated splicing events involving 1526 genes and 3200 RFS associated splicing events involving 1916 genes were uncovered (Supplementary Table 3). There are 670 intersections between the splicing events associated with OS and RFS, involving 656 genes (Fig. 3a, b), suggesting significant consistency between the splicing events associated with OS and RFS. Splicing events associated with OS are shown in Fig. 3c. It can be seen from the figure that the ES events is not strongly associated with OS. By contrast, the AT events appear to be strongly associated to OS. This pattern of results are also found when analyses were carried with RFS (Fig. 3d), suggesting that most of the ES events were not associated with prognosis (about 3.8% associated with OS and 4.8% associated with RFS), while approximately 10% of the AT events were significantly associated with prognosis.

**Figure 4:**
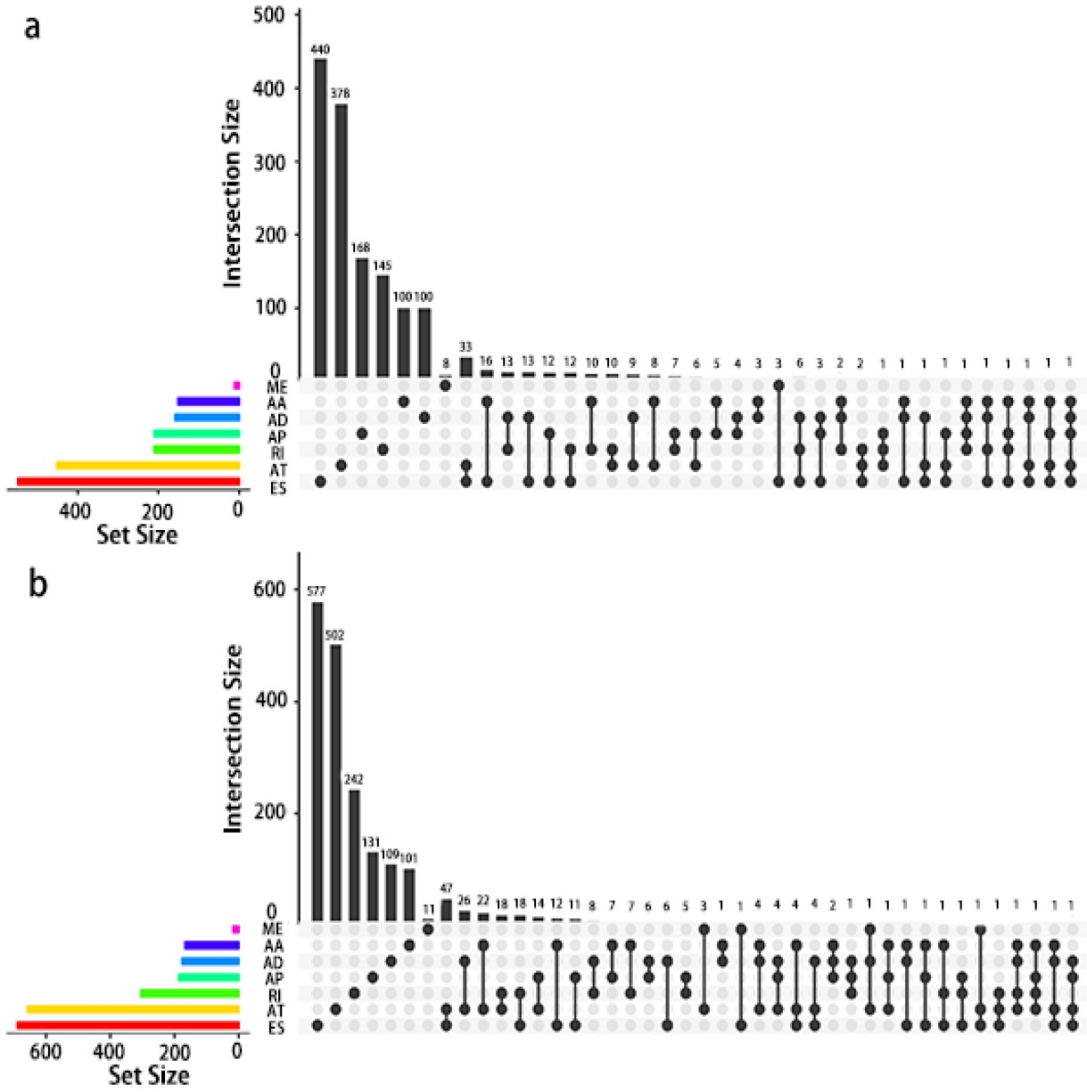
** UpSet plot of AS in PCa.** a, UpSet plot of interactions between the seven types of OS associated AS events. One gene may have up to five types of AS to be associated with patient survival. b, UpSet plot of interactions between the seven types of RFS associated AS events. One gene may have up to four types of AS to be associated with patient recurrence.

Given that a single gene may possess more than two alternative splicing patterns that may be strongly related to prognosis, an UpSet plot was drawn to better visualize the intersecting functions. This diagram is depicted in Fig. 4.

### Potentials of AS to predict PCa prognosis

**Figure 5:**
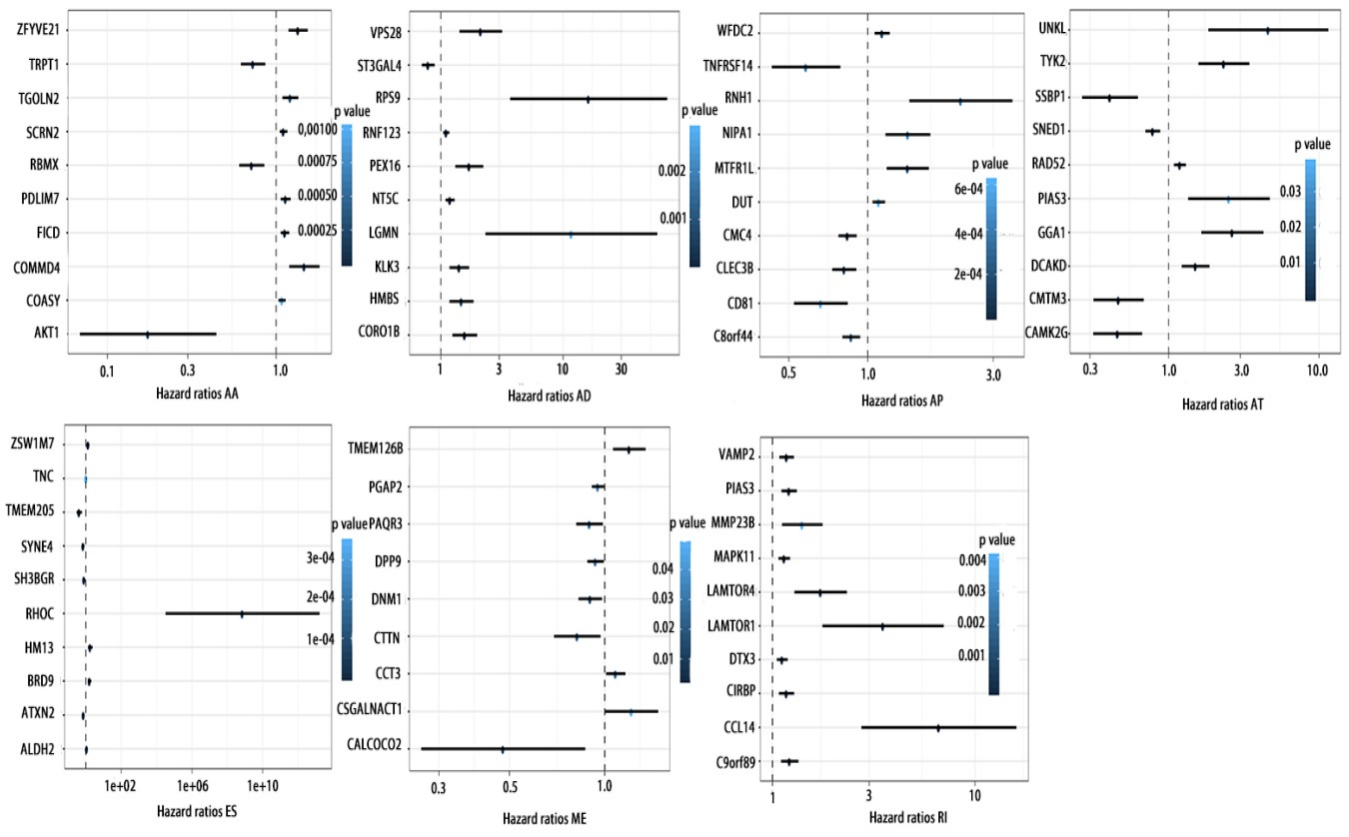
** Forrest plots of top10 OS associated AS events in PCa**. Hazard ratios of top10 OS associated AA, AD, AP, AT, AD, ES, ME and RI events. P values were indicated by color scale by the side.

**Figure 6:**
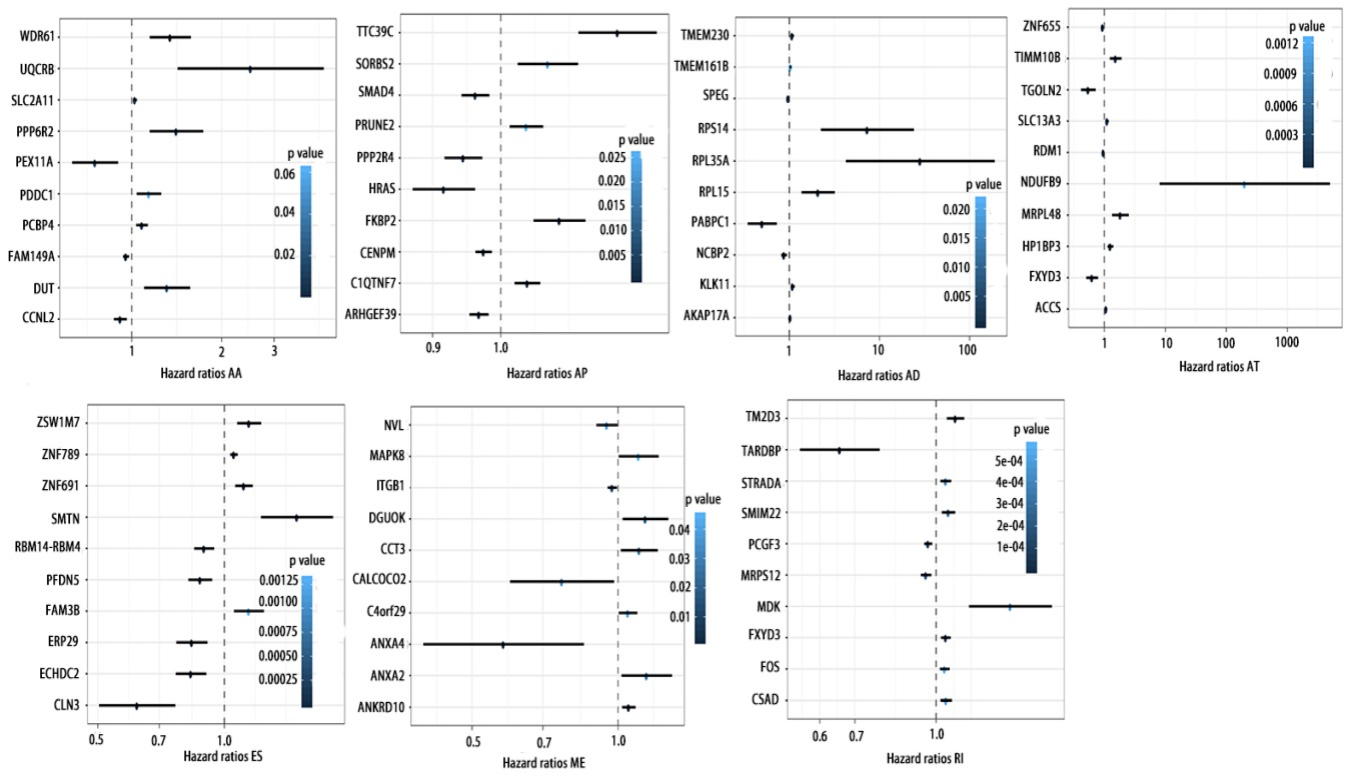
** Forrest plots of top10 RFS associated AS events in PCa**. Hazard ratios of top10 RFS associated AA, AD, AP, AT, AD, ES, ME and RI events. P values were indicated by color scale by the side.

**Figure 7:**
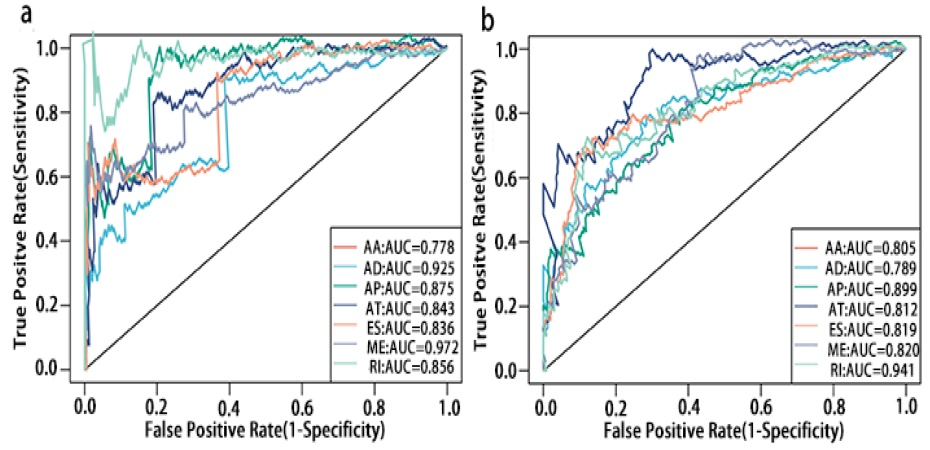
** ROC curves of top10 prognosis associated AS for PCa patients**. a, ROC curves with AUCs of top10 OS associated AS built by seven types of AS events. b, ROC curves with AUCs of top10 RFS associated AS built by seven types of AS events.

To detect whether the selected variable shear event can be used as a prognostic signature, the top 10 most significant prognosis associated alternatively spliced genes across seven types of AS are illustrated in Fig. 5 and Fig. 6. Kaplan-Meier analysis was applied (Supplementary Figure 1, 2), followed by multivariate Cox regression in order to generate a risk score system comprising of potentially prognostic alternatively spliced genes. ROC curve analyses were generated to assess the sensitivity and specificity of gene signatures and to estimate the discriminatory power of the prognostic gene expression signatures (Computational formulas are provided in Supplementary Table 4). The AUC was calculated and the data showed that all seven types of splicing events exhibited high efficiency with a large AUC, while ME type events were associated with the most optimal OS (AUC: 0,972) and RI type events associated with the most optimal RFS (AUC: 0.941) (Fig. 7).

### Gene networks prognosis- associated alternative splicing events and functional enrichment analyses

**Figure 8:**
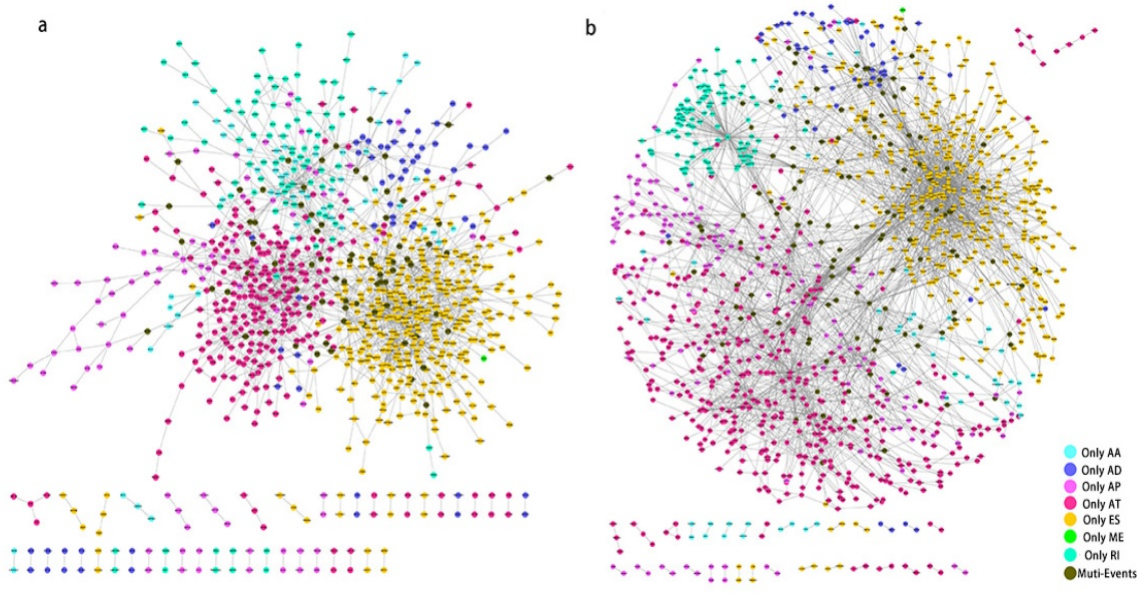
** PPI network of prognosis associated alternatively spliced genes in PCa.** a, PPI network of OS associated alternatively spliced genes. b, PPI network of RFS associated alternatively spliced genes.

**Figure 9:**
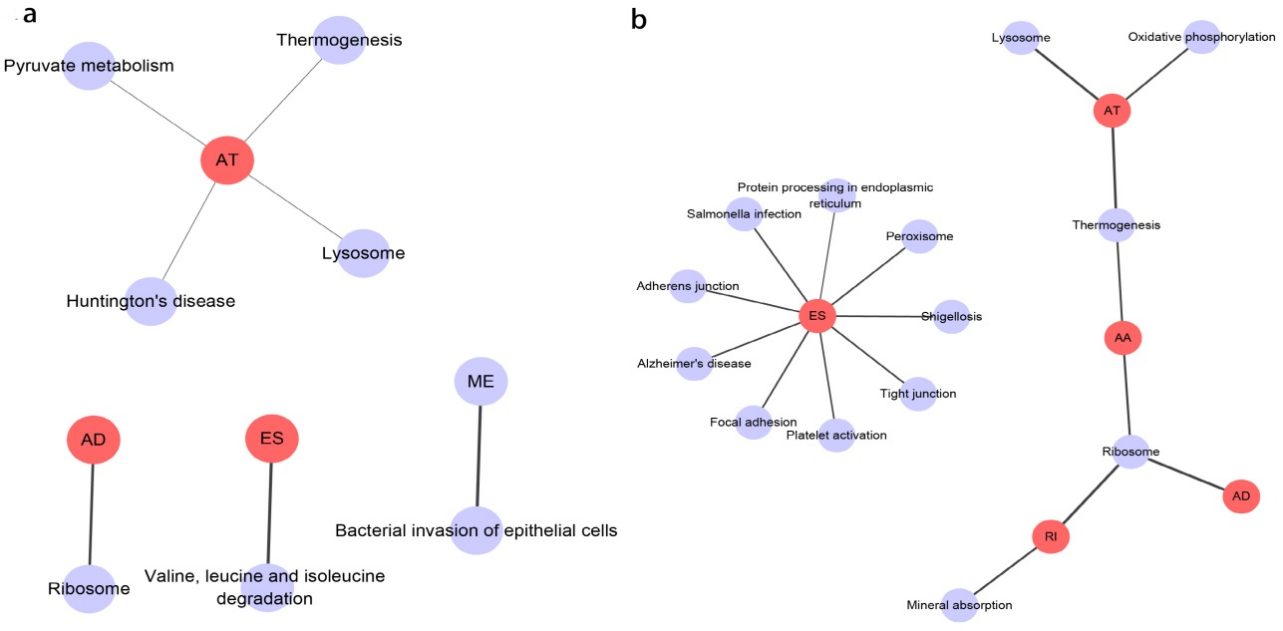
** KEGG analysis result of the survival outcomes associated alternatively spliced genes.** a, KEGG analysis result of the OS associated alternatively spliced genes. b, KEGG analysis result of the RFS associated alternatively spliced genes.

Prognosis associated alternatively spliced genes were computed into STRING for characterizing the gene interaction networks. These were then visualized using Cytoscape (Fig. 8). We documented the regulatory network regulating splicing with their respective significant OS and RFS associated alternatively spliced genes. As shown in Fig. 8, AT and ES participated in most of the interactions. We also calculated the number of alternatively spliced genes in PPI for each AS event (Table 1) and observed that most of the prognosis associated alternatively spliced genes participated in protein interactions, suggesting that a majority of the genes involved function to regulate different biological functions. To further gain insight into the biological roles of the alternative spliced genes, we performed KEGG pathway analyses. 7 KEGG enriched pathways associated with OS including AT: pyruvate metabolism, Huntington's disease, lysosome, thermogenesis, ME: bacterial invasion of epithelial cells, AD: ribosome, ES: valine, leucine and isoleucine degradation. 14 KEGG enriched pathways associated with RFS including ES: salmonella infection, protein processing in the endoplasmic reticulum, peroxisome, adherens junction, shigellosis, Alzheimer's disease, tight junction, focal adhesion, platelet activation, AT: lysosome, oxidative phosphorylation, thermogenesis, AA: thermogenesis, ribosome, RI: mineral absorption, ribosome, AD: ribosome. Importantly, survival associated AT was found to be a critical splicing event in OS and participates in 4 pathways while survival associated ES played a major role in RFS as it was involved in 9 pathways. Additionally, AA, AD, and RI were enriched in the ribosome pathway, indicating this pathway may correlate with recurrence (Fig. 9).

### The relationship between AS events associated genes expression profile and prognosis and screening of prognostic signature

Furthermore, univariate survival test was also conducted with genes expression profile involving in splicing events as identified above. As shown in supplementary Table 5, the expression of 396 genes among 1526 OS associated alternatively spliced genes were associated with OS. Similarly, 483 genes among 1916 RFS associated alternatively spliced genes were associated with RFS (Supplementary Table 6). Associations between the PSI prognostic values and gene expression levels of prognosis associated alternatively spliced genes were evaluated with the Spearman's test. Finally, we obtained 237 (59.8%) genes significantly correlated with OS associated alternatively spliced genes (*p* <0.05), and 329 (68.1%) genes significantly correlated with RFS associated alternatively spliced genes (Supplementary Table 7, 8).

### Screening establishment of prognostic scoring system based on six-gene signatures

**Figure 10:**
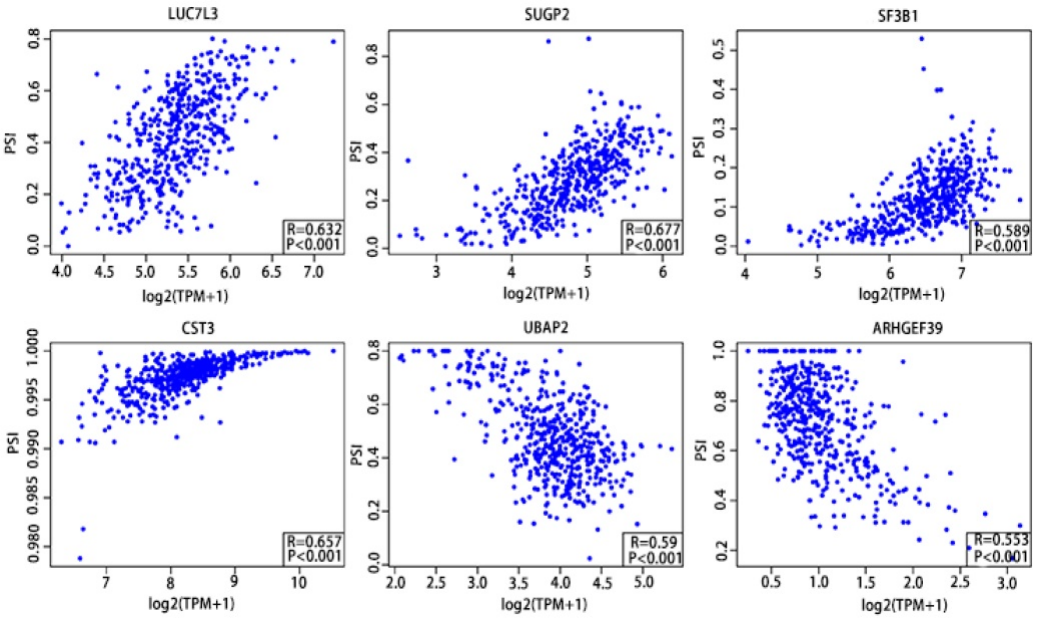
Pearson correlation analysis of the 6 candidate alternatively spliced genes and the AS.

As previously described, the Pearson correlation coefficient between candidate alternatively spliced genes and AS events was calculated, selecting genes with |Pearson correlation coefficient| >0.50 as candidates to construct the prognostic signature for PCa patients. There were 46 genes associated with RFS (Supplementary Table 9,10) and 7 associated with OS (Supplementary Table 11,12), with 6 genes overlapping from the two groups (Supplementary Table 13,14). These six genes were then utilized to construct a prognostic risk scoring system for PCa. Among these, LUC7L3, SUGP2, SF3B1 and CST3 showed positive coefficients in Pearson correlation analysis. For the remaining two genes, we observed negative coefficients in Pearson correlation analysis (Fig. 10).

The computational formulas are provided as follows:

Data of OS associated gene expression: Risk score= (0.0659*express level of LUC7L3) + (-0.043*express level of SUGP2) + (-0.013*express level of SF3B1) +(0.0004*express level of CST3) + (0.197*express level of UBAP2) +(0.114*express level of ARHGEF39)

Data of RFS associated gene expression: Risk score= (0.006*express level of LUC7L3) + (0.023*express level of SUGP2) +(-0.009*express level of SF3B1) + (-0.002*express level of CST3) + (0.059*express level of UBAP2) +(0.150*express level of ARHGEF39)

Data of OS associated PSI: Risk score= (0.021*PSI of LUC7L3) + (0.029*PSI of SUGP2) + (0.016*PSI of SF3B1) + (-1.892*PSI of CST3) + (-0.052*PSI of UBAP2) +(-0.028*PSI of ARHGEF39)

Data of RFS associated PSI: Risk score= (0.0009*PSI of LUC7L3) + (0.0182*PSI of SUGP2) + (0.0122*PSI of SF3B1) + (-0.6707*PSI of CST3) + (0.002*PSI of UBAP2) + (-0.0319*PSI of ARHGEF39)

**Figure 11:**
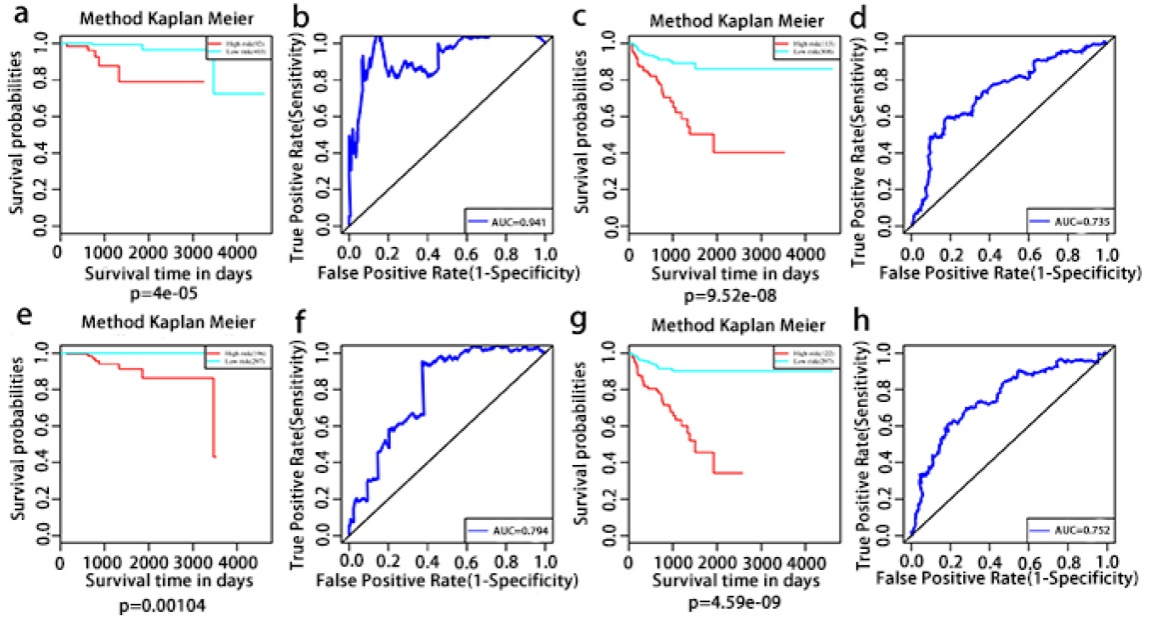
** Kaplan-Meier and ROC curves of prognosis risk score system for PCa patients.** a, Kaplan-Meier curves of OS associated AS events. c, Kaplan-Meier curves of OS associated gene expressions. e, Kaplan-Meier curves of RFS associated AS events. g, Kaplan-Meier curves of RFS associated gene expressions (red line indicates high risk group while the blue line indicates low risk group). b, ROC curves of OS associated AS events. d, ROC curves of OS associated gene expressions. f, ROC curves of RFS associated AS events. h, ROC curves of RFS associated gene expressions.

The multivariate Kaplan-Meier survival curves of the two groups based on the score system notably different, showing good performance in distinguishing either OS or RFS for patients with high-risk and low-risk (Fig. 11a, c, e, g). The effectiveness of this prognostic model was confirmed using ROC curves. The data showed that the prognostic risk score system (OS associated AS events) exhibited the highest efficiency with an AUC of 0.941, followed by risk score (OS associated gene expressions) with an AUC of 0.794, risk score (recurrence-free survival associated gene expressions) with an AUC of 0.752 and risk score (recurrence-free survival associated AS events) with an AUC of 0.735.

### Confirmation of the six gene expression

**Figure 12:**
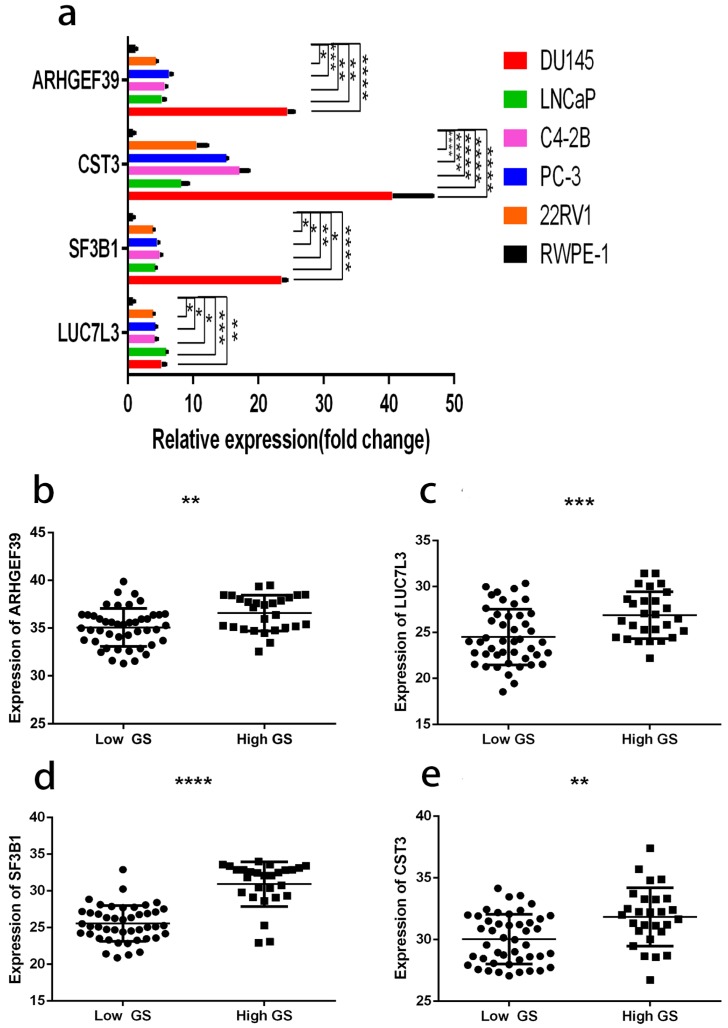
a, gene expression profiles in different cell lines. b, ARHGEF39 expression profiles in clinical samples. c, LUC7L3 expression profiles in clinical samples. d, SF3B1 expression profiles in clinical samples e, CST3 expression profiles in clinical samples. In clinical samples, the target gene expression was normalized to β-actin (ΔCt) and compared with the maximum ΔCt. Data are presented as ΔΔCt. Results are presented as mean ± SD. *p <0.05, **p < 0.01, ***p < 0.001, ****p < 0.0001.

As shown in Fig. 12a, the expressions of these 6 genes were first confirmed in prostate cell lines. We found that the expression of four among these six genes increased from normal prostate epithelial to PCa in situ and metastatic PCa. These four genes were also consistently raised in the clinical samples (Fig. 12b, c, d, e). The clinical samples contain 27 high and 45 low grade tumors. The lowest gene expression value was used in order to allow direct data comparison.

## Discussion

PCa is known for its biological heterogeneity which plays a role in the development of castration-resistant prostate cancer (CRPC), posing a substantial obstacle for physicians. Alternative pre-mRNA splicing may be a key genetic process which could open up new possibilities for developing novel markers for early diagnosis or optimal risk stratification, and targets for future therapeutic approaches 5,18. Preliminary investigations into the utility of aberrant AS event in predicting patient outcome has been widely documented in recent literature. For example, androgen receptor-variants (AR-Vs) are unusually shortened AR protein isoforms that retains the NH2-terminal domain but lacks the variable COOH-terminal domains which encompasses the ligand binding domain (LBD) 19. Several preclinical investigations have suggested that AR-Vs, specifically AR-V7, the most frequently expressed AR-V, may be linked to the development of enzalutamide and abiraterone resistance 20. Additional efforts are underway to develop selective AR-V inhibitors, which is a critical in augmenting current chemotherapeutic options in those with CRPC. Additionally, a classic tumor suppressor gene KLF6, Kruppel-like factor 6 splice variant 1 (KLF6-SV1) splice variant displays a markedly contrasting effect on tumors. Raised expression of KLF6-SV1 is associated with poorer prognosis and disease recurrence in several cancers including PCa 21.

As mentioned above, in PCa, the function or prognostic values of single alternative splicing events of genes have been well studied, but no systematic identification and prognostic value analysis of splicing events have been reported. Several TCGA data-based research has been published. Genome-wide analysis and prognostic models of splicing events with TCGA RNA-seq data based on SASEs were performed and created in order to prognosticate patients with non-small cell lung cancer 6, colorectal cancer 7, bladder urothelial carcinoma 8, and ovarian cancer 22. In our current study, we provide a comprehensive analysis of alternative splicing events and risk scores based on SASEs in 495 PCa patients for predicting the prognosis of PCa patients. 44070 AS events from 10,381 genes were determined with more than half of the events being ES events. Univariate survival tests were conducted for both RFS and OS. There is consistency between the OS associated splicing events and RFS associated splicing events, which is in line with clinical evidence that the two outcomes share a similar tumorigenic process. Moreover, associations between the PSI values of prognosis associated alternative splicing events and gene expression levels of prognosis associated splicing factors were also highlighted in our analysis in order to more accurately predict prognostic associated alternative events. Eventually, we built the six-gene prognosis risk score system by correlation analysis for PSI and mRNA expression. The model shows satisfied AUC of ROC of both genes associated with survival and of those linked to PSI associated survival, suggesting these genes show promising potential in their application in PCa prognostication, as they can be detected more sensitively and may be utilized in the clinic.

The role of SF3B1 in cancer has been well studied. SF3B1 mutations have been described as driver mutation in several myeloid malignancies including chronic lymphocytic leukemia, chronic myelomonocytic leukemia and myelodysplastic syndrome 23,24. Additionally, a similar conclusion was also drawn for breast cancer 25, liver cancer 26 and uveal melanoma 27. ARHGEF39, which is a Dbl-family guanine nucleotide exchange factor, is implicated in the development of a range of malignancies such as gastric cancer 28 and non-small cell lung cancer 29. LUC7L3 belongs to the Luc7 family, is involved in RNA splicing and is used to identify MITF/TFE partners in translocation renal cell carcinoma 30. UBAP2 which codes for ubiquitin-associated protein 2, may be interlinked with 9p13.3 chromosomal region augmentation in prostate cancer, potentially participating in tumorigenesis 31. CST3 is a cysteine protease inhibitor that mediates tissue inflammation, neutrophil chemotaxis and bone resorption. Additionally, the association between CST3 and cancer progression was also been reported 32. In patients with melanoma, increased CST3 serum level was correlated with the higher metastatic rate 33. Janko Kos et al. reported that increased CST3 level was significantly correlated with poor survival in patients with colorectal cancer 34. However, SUGP2 have not yet been studied in cancer.

Further *in vivo* and *in vitro* experiments are required to confirm the significance of observed AS events in this study. The noted importance of regulation of these AS would also benefit from more experiments concerning the discovery of in-depth molecular mechanisms.

Patients in this cohort were stratified based on their Gleason score. Several different classification systems are currently being investigated, as it has been found that several factors may contribute towards PCa prognostication 35. Given the use of only Gleason score in this study, it is possible that other factors may have been overlooked.

In summary, we have provided a comprehensive investigation of splicing events in patients with PCa and identified prognosis associated AS events. We also screened the genes whose expression profile was significantly associated with prognosis. Finally, we constructed the prognosis risk score system based on six-gene signatures which functioned well to stratify risk for PCa patients. This work not only provided insight into the pathobiological means of AS in PCa tumorigenesis, but also lay the foundation for development of potential prognostic biomarkers and novel therapeutic targets of PCa.

## Supplementary Material

Supplementary figures.

Supplementary tables.

## Figures and Tables

**Table 1:** the number of alternatively spliced genes in PPI for each AS event.

	OS			RFS		
	Number of the genes in PPI network	Number of all genes	Percent	Number of the genes in PPI network	Number of all genes	Percent
AA	72	151	0.48	74	167	0.44
AD	79	159	0.50	88	176	0.50
AP	98	211	0.46	176	304	0.58
AT	288	452	0.64	496	688	0.72
ES	392	548	0.72	501	656	0.76
ME	3	11	0.27	2	17	0.12
RI	112	211	0.53	131	186	0.70
