# Supplementary material for: Comprehensive investigation of alternative splicing and development of a prognostic risk score for prostate cancer based on six-gene signatures
Source: J Cancer. 2019 Sep 7;10(22):5585–96. doi: 10.7150/jca.31725 (PMC6775697; doi:10.7150/jca.31725)
Supplement: Supplementary file 1 — Supplementary figures. [file jcav10p5585s1.pdf]

Supplementary Figure 1 Kaplan-Meier curves of top10 OS associated AS events in PCa. a to g, Kaplan-Meier curves of top10 OS associated AA, AD, AP, AT, ES, ME and RI events.

Supplementary Figure 2 Kaplan-Meier curves of top10 RFS associated AS events in PCa. a to g, Kaplan-Meier curves of top10 RFS associated AA, AD, AP, AT, ES, ME and RI events.

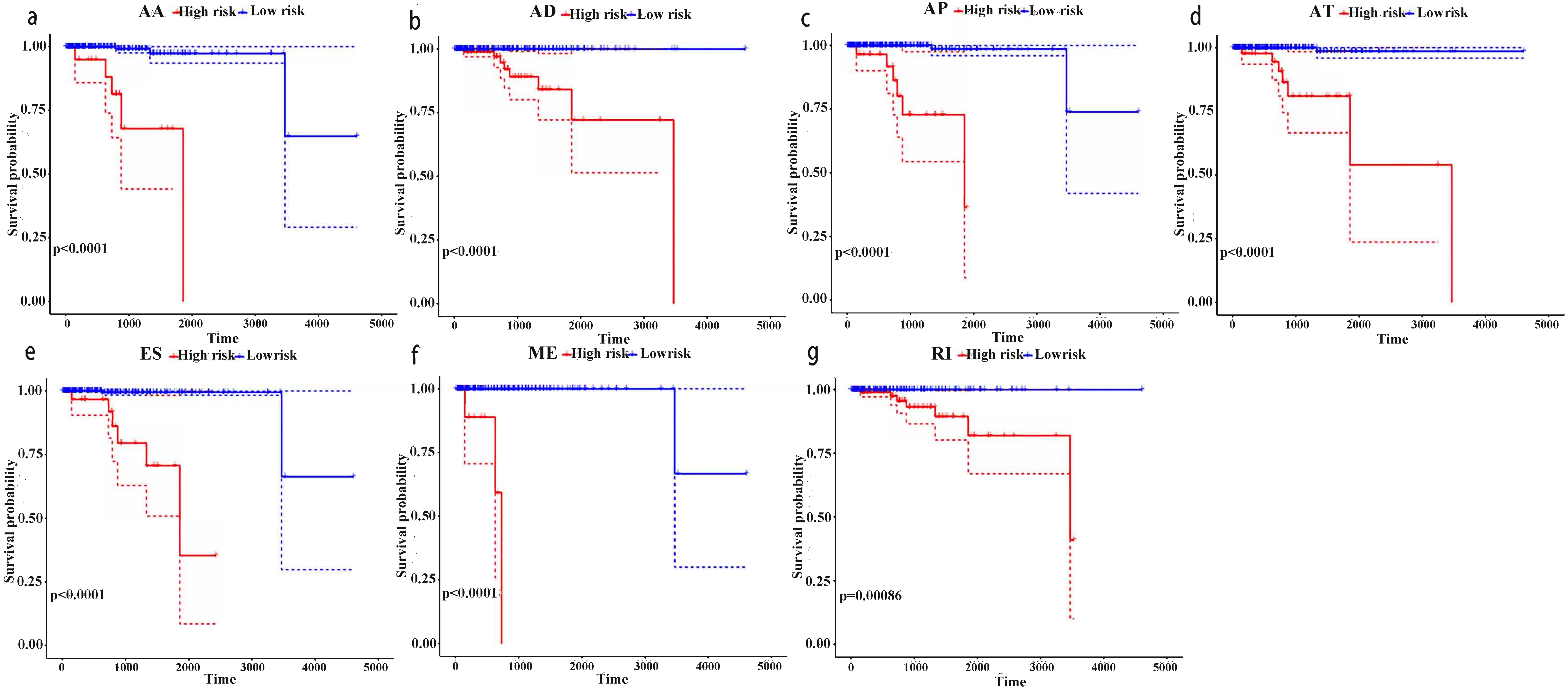

Fig. S1 Kaplan-Meier analysis of top10 OS associated AS events in PCa.

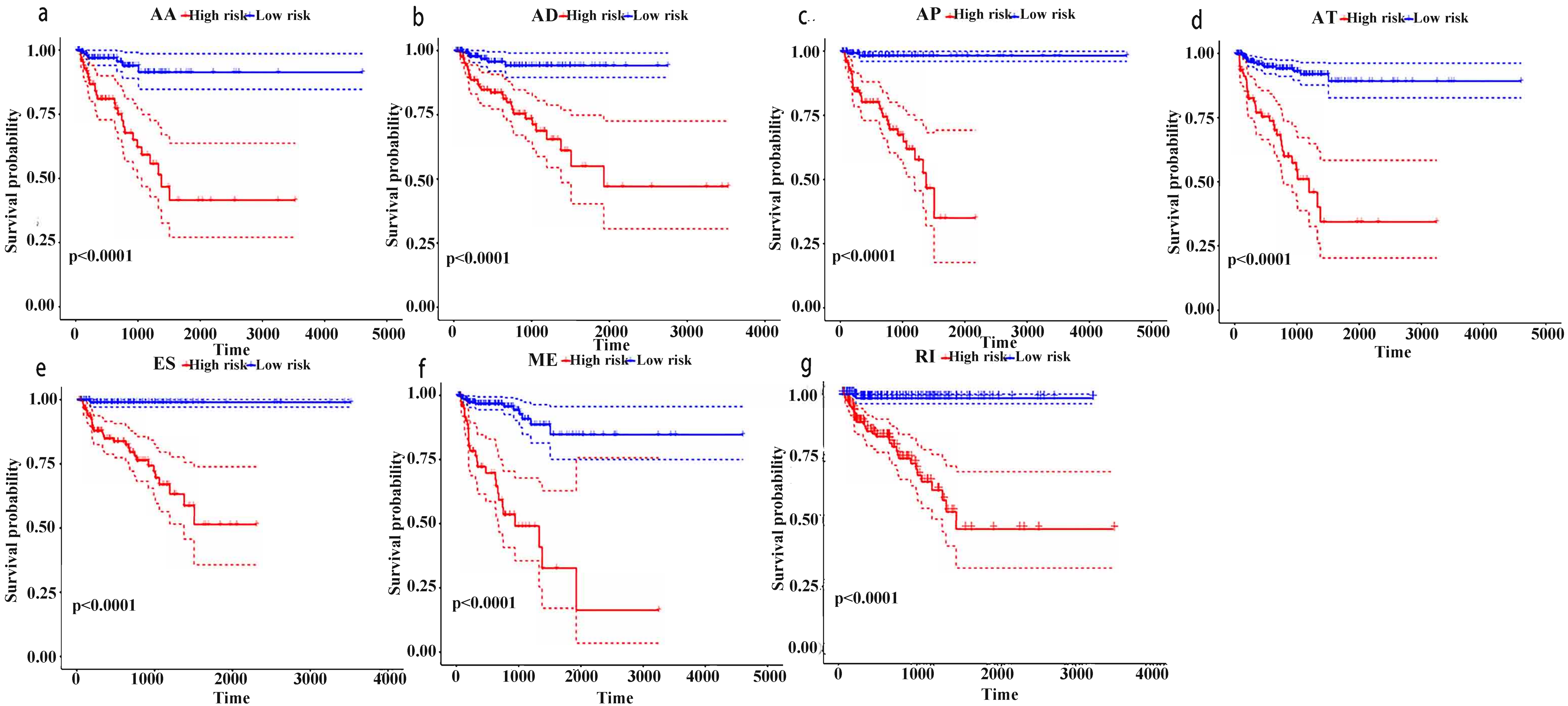

Fig. S2 Kaplan-Meier analysis of top10 RFS associated AS events in PCa.
